# Triglyceride-Fasting Glucose Index and Homeostatic Model Assessment for Insulin Resistance as Predictors of Type 2 Diabetes Mellitus in South Indians With Normal Body Mass Index

**DOI:** 10.7759/cureus.62742

**Published:** 2024-06-20

**Authors:** Jayashankar CA, Amey Joshi, Mohammed Ishaq, Gurucharan Adoor, Mahesh V, Harshavardhan Jampugumpula, Kavitha R, Bhangdiya G Sanjay, Prafulla K Bhupathiraju

**Affiliations:** 1 Internal Medicine, Vydehi Institute of Medical Sciences and Research Centre, Bangalore, IND; 2 Internal Medicine, University of Michigan Health-Sparrow, Lansing, USA; 3 Community and Family Medicine, Chamarajanagar Institute of Medical Sciences, Chamarajanagar, IND; 4 Internal Medicine, Manipal Hospital, Bangalore, IND; 5 General Medicine, Vydehi Institute of Medical Sciences and Research Centre, Bangalore, IND

**Keywords:** endocrinology and diabetes, homa-ir, tyg, type 2 diabetes mellitus, insulin resistance

## Abstract

Introduction: Early detection of type 2 diabetes mellitus (T2DM) is imperative to prevent the complications associated with the disease. Current guidelines for diagnosis rely on the assessment of serum glucose (fasting and post-prandial) and glycosylated hemoglobin (HbA1c) levels. Insulin resistance, a phenomenon associated with T2DM, has been observed before the changes in these metrics. The homeostatic model assessment for insulin resistance (HOMA-IR) has been widely used to assess the degree of insulin resistance. The triglyceride-fasting glucose (TyG) index is a newer marker of insulin resistance that merits further study.

Aim: The study aimed to assess the validity of the TyG index and HOMA-IR as markers for the development of T2DM in non-obese individuals.

Materials and methods: One hundred eight non-obese patients without T2DM were included in this prospective cohort study and followed up for eight years. Anthropometric and biochemical parameters, including fasting glucose levels, HbA1c, fasting serum insulin, low-density lipoprotein (LDL), high-density lipoprotein (HDL), and triglycerides (TG), were measured at enrolment and eight years follow-up, and HOMA-IR and TyG index were calculated.

Results: Twenty participants out of 108 (18.5%) developed T2DM over eight years. On performing the area under the curve (AUC)-receiver operating characteristic curve analysis, TyG of >8.61 and HOMA-IR of >1.5 had the highest validity (ability) to predict new-onset T2DM in the study population (TyG: AUC: 0.612 (95% CI: 0.514-0.705); HOMA-IR: AUC: 0.529 (95% CI: 0.431-0.626)); however, this was not statistically significant.

Conclusion: At an eight-year follow-up, TyG and HOMA-IR were unreliable predictors of the development of T2DM in non-obese individuals.

## Introduction

Diabetes mellitus affects over 170 million individuals worldwide, and the prevalence of this disease is estimated to increase to over 365 million individuals by 2030 [1]. Untreated or poorly managed diabetes can lead to microvascular and macrovascular complications, including cardiovascular disease, renal disease, neuropathy, blindness, and amputation. Early detection through screening can help healthcare providers develop a personalized treatment plan that includes lifestyle changes, medication, and regular follow-up to prevent or delay the onset of disease complications, thereby improving the individual's quality of life and reducing healthcare costs.

Insulin resistance is one of the main pathophysiological mechanisms leading to the development of type 2 diabetes mellitus (T2DM) and has been found to precede the diagnosis of the disease by at least 10 years [2,3]. Diagnosing insulin resistance in individuals may help healthcare providers stratify patient populations based on risk assessment and adapt treatment interventions and follow-up even before the diagnosis of T2DM. The homeostatic model assessment for insulin resistance (HOMA-IR) is an indirect marker of insulin resistance and is most widely employed in clinical practice because of its convenience [4]. However, the high cost and limited availability of serum insulin limit its use in wider clinical settings. The triglyceride-glucose (TyG) index is an accessible and reliable surrogate marker of insulin resistance. It has been shown to have comparable performance to HOMA-IR in individuals with and without T2DM [5]. Furthermore, the TyG index does not require insulin quantification and is unaffected by exogenous insulin administration. 

However, the validity of the TyG index as a predictor for the development of T2DM remains to be established in a non-obese population principally investigated in South India. The present study aims to compare HOMA-IR and TyG index as predictors of T2DM at eight-year follow-up in non-diabetic and non-obese individuals.

## Materials and methods

Methods

This prospective cohort study was conducted between January 2014 and December 2022 at Vydehi Institute of Medical Sciences and Research Centre in Bangalore, Karnataka, India. A total of 108 non-obese participants were enrolled after obtaining written informed consent. Clinical data, including demographic details and laboratory data (fasting blood sugar (FBS), glycosylated hemoglobin (HbA1c), fasting serum insulin, low-density lipoprotein (LDL), high-density lipoprotein (HDL), and serum triglycerides (TG)), was collected at the time of enrolment and eight-year follow-up. 

Patients who were >18 years of age and with a body mass index (BMI) <22.5 kg/m^2^ were included in the study. Participants with a history of diabetes mellitus, dyslipidemia, hypertension, fatty liver disease, cardiovascular disease, chronic obstructive pulmonary disease (COPD), chronic kidney disease (CKD), and thyroid disease were excluded from the study. Fasting blood glucose, glycosylated HbA1c, serum creatinine, and thyroid-stimulating hormone were tested prior to participant recruitment. Patients with a history of chronic alcoholism and who were lost to follow-up were also excluded from the study. At the time of enrolment, participants were screened for T2DM as per the American Diabetes Association (ADA) criteria by two-hour glucose levels ≥200 mg/dl during an oral glucose tolerance test (OGTT), fasting serum glucose 126 mg/dl, or HbA1c 6.5% [6]. The Institutional Review Board of Vydehi Institute of Medical Sciences and Research Centre approved the present study (approval number: VIEC/2013/APP/154). 

Height was measured using a stadiometer (meter), and weight was measured using a digital scale (kilogram). The BMI was calculated by dividing the weight by height squared (kg/m^2^). HOMA-IR was calculated using the following formula: (fasting insulin (µU/ml) × fasting plasma glucose (mg/dl))/405 [7]. TyG index was calculated using the following formula: ln (fasting triglycerides (mg/dl) × fasting plasma glucose (mg/dl)/2) [8]. 

Statistical analysis

Using the IBM SPSS Statistics for Windows, V. 23.0 (IBM Corp., Armonk, NY, USA), statistical analysis was performed, and the collected data were analyzed. Categorical data was represented in the form of frequencies and proportions. Continuous data were expressed as mean and standard deviation. The normality of the continuous data was tested by the Kolmogorov-Smirnov test and the Shapiro-Wilk test. Analysis of variance (ANOVA) was the test of significance to identify the mean difference between more than two groups for quantitative data. The receiver operating characteristic (ROC) curve analysis was performed to assess the predictivity of the markers in the study and determine cut-off values. A p-value of <0.05 was considered statistically significant. 

## Results

Clinical characteristics of the study population 

The clinical characteristics of the study population with biochemistry parameters are presented in Table 1. Twenty of the 108 participants developed T2DM at an eight-year follow-up with an incidence rate of 18.5%. A significant increase in fasting plasma glucose (FPG) compared to the initial visit FPG at the eight-year follow-up was noted (85.80 ± 6.986 mg/dl at the initial visit to 144.550 ± 18.613 mg/dl at eight years). The mean age of the participants at enrolment was 39.51 ± 12.64, and the mean BMI was 20.96 ± 1.36. The mean HOMA-IR and TyG index during the enrolment period were 2.06 ± 1.08 and 8.61 ± 0.26, respectively. HOMA-IR and TyG index had negligible correlation by Pearson correlation analysis (P=0.130). 

**Table 1:** Baseline characteristics BMI: body mass index; HOMA-IR: homeostatic model assessment for insulin resistance; HDL: high-density lipoprotein; LDL: low-density lipoprotein; VLDL: very-low-density lipoprotein; TyG: triglyceride-glucose

Baseline characteristics	Mean	SD	Median
Age (years)	39.51	12.64	38
Height (m)	1.54	.06	1.55
Weight (kg)	49.66	4.01	49
BMI	20.96	1.36	21.05
Fasting plasma glucose (mg/dl)	85.00	8.88	85
Fasting serum insulin (uIU/ml)	9.86	5.31	8.70
HOMA-IR	2.06	1.08	1.90
Serum total cholesterol (mg/dl)	163.12	20.22	163
Serum triglycerides (mg/dl)	132.84	36.18	132
Serum HDL cholesterol (mg/dl)	45.52	8.08	45
Serum LDL cholesterol (mg/dl)	95.19	16.77	94
Serum VLDL cholesterol (mg/dl)	26.82	9.48	27
TyG index	8.61	0.26	8.60

Comparison of ROC curves between the TyG index and HOMA-IR

**Figure 1:**
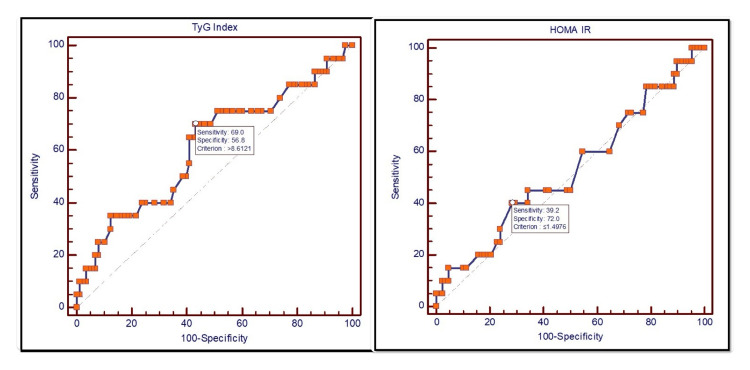
AUC by ROC of TyG index and HOMA-IR in T2DM AUC: area under the curve; ROC: receiver operating characteristic; HOMA-IR: homeostatic model assessment for insulin resistance; TyG: triglyceride-glucose; T2DM: type 2 diabetes mellitus

The area under the curve (AUC) for the TyG index and HOMA-IR was 0.612 (95% CI: 0.514-0.705) and 0.529 (95% CI: 0.431-0.626), respectively; however, this was not statistically significant (Table 2, Figure 1). TyG index performed better than HOMA-IR, but this difference was not statistically significant. HOMA-IR cut-off value >1.5 and TyG index cut-off value >8.61 provided a sensitivity and specificity of 40.00%, 71.59%, 70.00%, and 56.82%, respectively, in predicting T2DM onset within eight years duration. 

**Table 2:** AUC by ROC of TyG index and HOMA-IR in T2DM AUC: area under the curve; ROC: receiver operating characteristic; HOMA-IR: homeostatic model assessment for insulin resistance; TyG: triglyceride-glucose; T2DM: type 2 diabetes mellitus

	TyG	HOMA-IR
AUC by ROC	0.612	0.529
Standard error	0.0756	0.0757
95% confidence interval	0.514-0.705	0.431-0.626
z statistic	1.488	0.387
Significance level p (area = 0.5)	0.1367	0.6991
Youden's index J	0.2682	0.1159
Associated criterion	>8.61	≤1.5

## Discussion

In the present study, TyG and HOMA-IR had an independent and positive correlation with T2DM onset; however, their validity as predictors of T2DM onset for non-obese individuals at eight years was poor and statistically insignificant. TyG index of >8.61 and HOMA-IR of >1.5 had the highest validity (ability) to predict new-onset T2DM in the study population (TyG: AUC: 0.612 (95% CI: 0.514-0.705); HOMA-IR: AUC: 0.529 (95% CI: 0.431-0.626)). The sensitivities and specificities for these cut-off values were 70% and 56.8% for the TyG index and 40% and 71.6% for HOMA-IR, respectively.

Insulin resistance causes altered glucose and lipid metabolism, resulting in chronic inflammation, oxidative stress, and endothelial dysfunction. Insulin resistance increases hepatic very-low-density lipoprotein (VLDL) and TyG synthesis, which is linked to the increased hepatic apo B-100 production. Insulin resistance can also increase hepatic TG lipase, which results in accelerated clearance of HDL. A major factor in increased TyG in insulin resistance is the accelerated rate of lipolysis of stored TyG-derived fatty acids from adipose tissues. Insulin resistance can also reduce VLDL breakdown, thereby increasing TyG. As evidenced through multiple mechanisms, insulin resistance can cause an increase in TyGs [9].

Insulin resistance is a primary risk factor for the development of T2DM. Current guidelines recommend screening for FBS, post-prandial blood sugar (PPBS), and HbA1c levels; however, changes in these metrics may be detected significantly later than the onset of insulin resistance. The association between insulin resistance and obesity is well established; however, some studies have shown that individuals with normal-range BMI can develop insulin resistance and, if untreated, can develop T2DM [10-12]. In the South Indian peninsula, insulin resistance has been observed even in undernourished individuals owing to antenatal insults, poor nutrition, and breastfeeding and weaning practices [13]. This association between non-obese individuals and insulin resistance makes T2DM risk stratification difficult for clinicians. Developing novel methods or markers to detect insulin resistance is paramount to reducing the incidence of T2DM and its related complications. TyG index was shown to be a reliable and superior predictor of T2DM compared to HOMA-IR in previous studies conducted in individuals in Southeast Asia and Spain [14-19]. Evidence supporting this association in the South Indian peninsula is lacking. The absence of correlation between HOMA-IR and TyG and T2DM development in the present study population may be due to racial, genetic, and nutritional differences compared to the Southeast Asian and Western populations. This finding remains to be validated through more extensive longitudinal studies.

Furthermore, the lack of association between these markers and T2DM in the present study may also be due to the limited statistical power of the study owing to its small sample size. TyG index has shown promising results and consistency over HOMA-IR in detecting insulin resistance. The affordability, accessibility, and reliability of this marker merit more extensive longitudinal studies in the Indian population. 

There are several limitations to our study. The small sample size of our study may contribute to the low statistical power of the study. Although participants were screened for comorbidities that may later affect glucose metabolism, their recruitment from a hospital setting may have introduced Berkson's bias. As the present study is a longitudinal cohort study, participants may have altered their behavior during the study period, leading to a Hawthorne effect. However, the prospective design, methodology, and variables in the study are key strengths that may allow for reproducibility on a larger scale. 

## Conclusions

The present study found TyG and HOMA-IR to be unreliable predictors for the development of T2DM in non-obese individuals over an eight-year follow-up period. While both TyG and HOMA-IR showed some discriminatory ability, their performance did not reach statistical significance in predicting new-onset T2DM. More extensive longitudinal studies in the South Indian population are required to ascertain their validity as markers of T2DM development. 
